# Students physical activity assessed by accelerometers and motivation for physical education during class: Should we consider lessons as a whole or only active periods?

**DOI:** 10.1371/journal.pone.0229046

**Published:** 2020-03-03

**Authors:** Julie Boiché, Marc Yangüez Escalera, Julien Chanal

**Affiliations:** 1 EPSYLON EA 4556, Univ. Montpellier, Univ Paul Valéry Montpellier 3, Montpellier, France; 2 Faculty of Psychology, University of Geneva, Geneva, Switzerland; 3 Distance Learning University, Brig, Switzerland; University of Kentucky, UNITED STATES

## Abstract

**Background:**

This study explores the links between measures of motivation based on Self-Determination Theory, and physical activity (PA) indicators assessed with accelerometers during physical education lessons.

**Methods:**

Questionnaires about motivation and psychological needs on the context of physical education were completed by 319 middle high school students, up to 5 times within a 2-year period; they were equipped with an accelerometer and videotaped during a physical education lesson. PA-related indicators were computed considering the entire duration of the lessons (strategy 1), but also retaining only active times during the lesson (strategy 2).

**Results:**

When the first strategy was used very few correlations emerged between motivation constructs and PA-related indicators. On the other hand, the second strategy was more effective to detect the link between motivation and students activity during class-time, in particular with sedentary time, moderate and vigorous PA.

**Conclusions:**

This study shed light on the importance of considering how the sequences of PA sessions should be coded, in order to link psychological phenomena with PA levels, as well as to provide a meaningful support for motivational hypotheses.

## 1. Introduction

It is well known that regular physical activity (PA) plays an important role on children’s development and health. Indeed, it is recommended for children and adolescents to be physically active at least 60 minutes per day on average [1]. The limitation of sedentary time (SED) represents an increasingly important public health issue [2]. Physical education (PE) classes present an opportunity for youth to accumulate a significant amount of PA and limit daily SED. However, not all young individuals may benefit equally from the traditional PE approach. In particular, it was suggested that motivation toward PE could significantly impact actual behaviors [3]. Self-determination Theory (SDT) currently represents one of the most frequently used models to examine the outcomes of students’ motivation [4]. Research previously showed the pertinence of this framework to understand PA-related behaviors and SED in PE classes [5]. However, in this study, we were also interested in adding a methodological perspective that could enable a better capture of the relationships investigated. Specifically, we tested these links according to different periods of the PE lessons and investigated if some differences occurred.

### 1.1. Self-determination theory (SDT)

**Fig 1:**
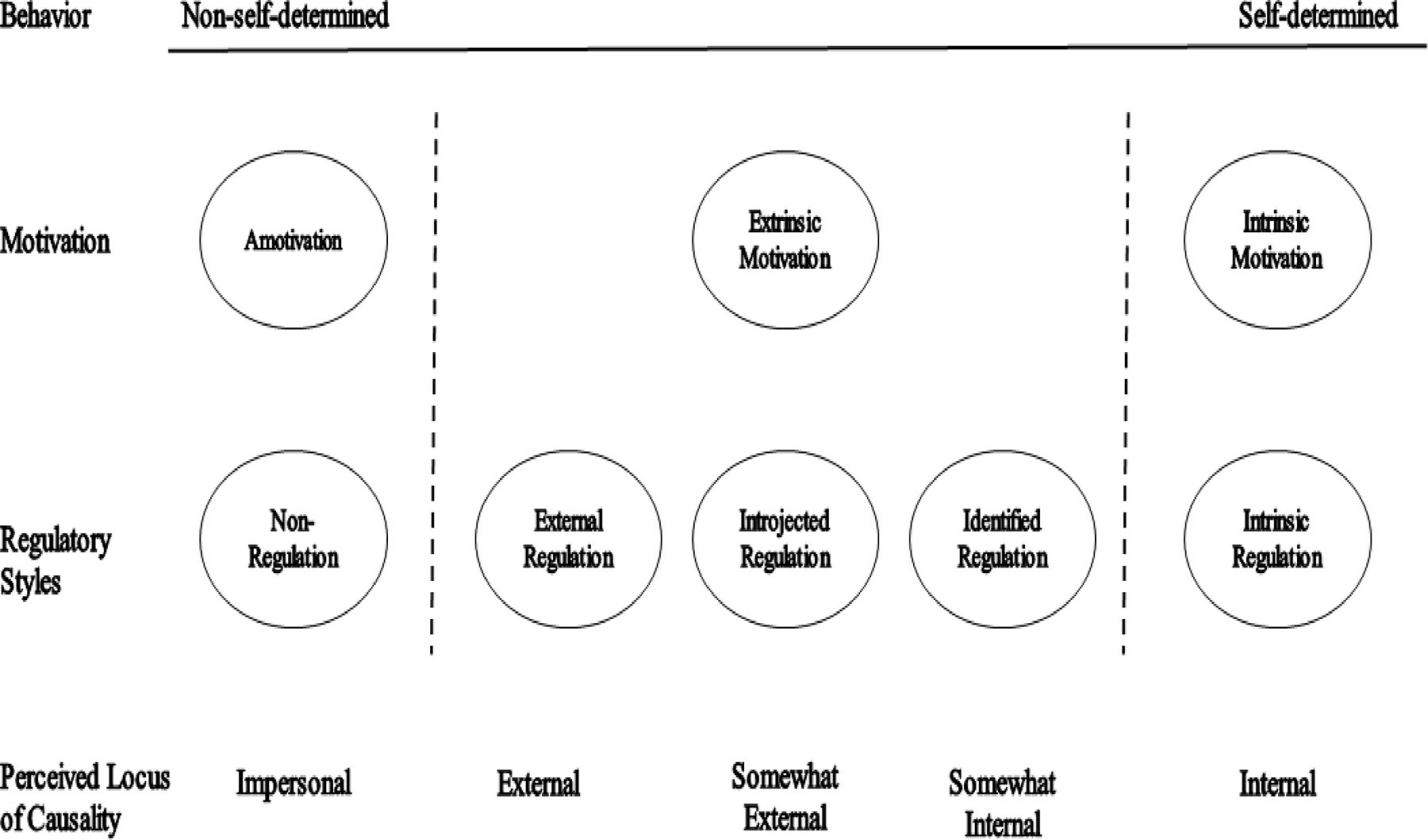
The taxonomy of regulatory styles. Adapted from Ryan & Deci (2000).

SDT posits that when executing particular behaviors, an individuals’ level of self-determination–i.e., perceived autonomy–or on the contrary constraint, is a key factor as to whether they will demonstrate more or fewer positive outcomes. The SDT continuum of motivation encompasses intrinsic motivation (i.e., doing something that in itself provides pleasure and satisfaction), various forms of extrinsic motivation (i.e., doing something not for its inherent interest, but to achieve a different purpose), and amotivation. From the most self-determined form to the least, extrinsic motivation can be categorized as identified regulation (i.e., adopting a behavior because it has been identified as a good means to reach a personally valued goal), introjected regulation (i.e., acting out of personal pressure), and external regulation (i.e., acting in relation to external contingencies) (see Fig 1). The last two forms of motivation can be conceptualized as the orientation toward attaining valued outcomes (e.g., to attain self-worth perceptions, or rewards from one’s environment) or avoidance (e.g., of negative affects related to oneself, or punishments from one’s entourage).

SDT also proposes hypotheses as to why certain forms of motivation may be preferentially developed by individuals in specific contexts. It assumes that motivation will be more self-determined, and hence outcomes will be more positive, if basic psychological needs are fulfilled. That is, the more individuals experience a great sense of autonomy (i.e., feeling that one has options and is at the origin of his/her actions), competence (i.e., feeling able to master one’s environment) and relatedness (i.e., feeling known and accepted by others), the more motivation will be self-determined [6]. While numerous studies have investigated potential outcomes of motivation in educational domains, previous work on behavioral aspects mostly interrogated persistence or achievement [4]. In the case of PE, no previous research was conducted on SED and there were few attempts to date to examine the relationship between motivational characteristics of students and their PA levels.

### 1.2. SDT motivation constructs and PA-related behaviors in PE lessons

If numerous studies were conducted to assess the link between PE motivation and general levels of PA in adolescents, there were few attempts to examine the is association using PA levels observed during the PE lessons. The research is even scarcer if one considers only studies that employed an objective assessment of PA, which is currently recommended considering the limitations of self-report scales [7]. Three previous studies used pedometers to account for this variable. Self-determination index was significantly associated with step-count in secondary school Hong-Kong students [8]. A more specific result was reported by Johnson et al. (2017) who observed a significant link between PE enjoyment and step counts in 6^th^ to 8^th^ grade US students [9]. On the other hand, no significant associations between motivations (intrinsic motivation, identified regulation, external regulation, amotivation) and PA were found in US students from 6^th^ to 8^th^ grade [10]. Two additional studies were conducted using accelerometers. Associations between motivation and moderate-to-vigorous PA were investigated in a sample of Australian 9^th^ graders [11]. Intrinsic motivation and identified regulation were positively related to PA levels, while external regulation was unrelated, and amotivation was negatively associated. Conversely, no relationships were found between those constructs in a sample of American elementary students [12]. To the best of our knowledge, there were no attempts in previous research to investigate the relationship between the satisfaction of psychological needs and PA levels during PE classes in primary school students. However, this parameter appears to be significantly related to objectively assessed PA levels at this age [13].

### 1.3. The present study

Using objective devices such as pedometers or accelerometers to measure PA-related behaviors is currently considered the gold standard of assessment. However, there is great variation in terms of methods of measurement of PA and the nature of the activity elements that estimate PA duration. For instance, some authors considered a 20-minute structure session of basketball led by the teacher [9]. Others researchers began recording PA only 20 minutes after students were changed [8]. In another study, total class time (from start of class to end of class) was considered [10]. Noteworthy, as stressed by Gao and colleagues (2013), a typical PE class is classically composed of a warm-up period, instructions, skill practice and game play, and a large percentage of time can be attributed to class management and instruction [14]. As such, children’s PA in PE is intermittent in nature and there are even sequences of lessons that are not deemed to generate movement. The relationships observed between psychological characteristics, such as motivation, and PA-related scores, is thus likely to vary depending on the measurement strategy employed.

In the current study, we explored the relationships between satisfaction of basic psychological needs in PE among students, as well as their motivations in this context, and the levels of PA-related behaviors displayed in PE lessons, assessed objectively with accelerometers. It was hypothesized that higher levels of autonomy, competence, relatedness, intrinsic motivation, and identified motivation, would be positively related to PA and negatively to SED, while introjected regulation, external regulation and amotivation would be negatively related to PA and positively to SED. Moreover, we considered different periods in the PE lesson to investigate these relationships. More precisely, we hypothesized that these relationships would vary throughout the lesson according to which periods could reflect more individual motivation. Specifically, we hypothesized that removing the collective and non-active lesson times period (i.e., sequences during which teachers give instructions or feedback and during which students are expected to stay motionless), would enable us to better capture students’ unique behaviors, thus better relating to motivation per se.

## 2. Materials and methods

### 2.1. Procedure and participants

The results presented are part of a large study conducted on motivation and physical activity in Swiss adolescents [15]. During this investigation, 40 PE lessons were filmed. Many different sport and physical activities were practiced (e.g. soccer, rugby, volleyball, badminton, and gymnastics). In the current study, we considered the data provided by students that wore an accelerometer during these 40 videotaped lessons. The sample thus involved 319 students in grade 6 to 8 (47% female; age range = 9–13). Throughout the duration of the entire study, questionnaires assessing motivation and psychological needs in PE were completed during regular classes on five different occasions.

The University of Geneva approved this research. In agreement with the Ethics Committee, all participants were given written informed consent to be signed by their parents prior to participation, and received a written debriefing at the end of the study. The data was obtained and analyzed anonymously.

### 2.2. Measures

#### 2.2.1. Motivation

Motivation toward PE was measured through a 33-item questionnaire divided into 8 sub-scales, based on the Behavioral Regulation in Exercise Questionnaire-23 [16], the Exercise Motivation Scale [17], and the Sport Motivation Scale [18].

A Confirmatory Factor Analysis sustained an 8-factor model and the distinction between intrinsic motivation-stimulation (α = .80), intrinsic motivation-accomplishment (α = .71), identified motivation (α = .80), introjected regulation-approach (α = .66), introjected regulation-avoidance (α = .74), external regulation-approach (α = .78), external regulation-avoidance (α = .83), and amotivation (α = .72).

#### 2.2.2. Psychological needs

Perceptions of autonomy, competence and relatedness were assessed with 5 items each with the Basic Psychological Needs Scales [19]. Internal consistency was satisfactory in the current sample (α = .88, α = .67, α = .76, for competence, autonomy and relatedness respectively).

We assessed motivation and psychological needs toward PE between two and five times for each student during the protocol and, considering that the lessons included different physical activities, we decided to compute the mean score of all the available measures we had for each student of the sample. This enabled us (1) to obtain scores that better reflect the PE motivations of the students, and to limit potential bias due to the variations of time, situations, or activities; (2) to prevent the reduction of the sample size considering correlations between PA-related behaviors with motivations and psychological needs.

#### 2.2.3. PA-related behaviors

Accelerometers were used to record the percentage of time that children spent doing moderate-to-vigorous PA (MVPA). Activity count cut-offs for 15-s epochs were applied: sedentary (i.e. ≤1.5 MET, ≤25 counts), light (i.e. >1.5 MET, > 26 and ≤ 3 MET, ≤ 573 counts), moderate (i.e. ≥3 MET, ≥574 and ≤ 6MET, ≤ 1002 counts), and vigorous intensity (i.e. ≥6 MET, ≥1003 counts per 15-s epochs). After the recordings were prepared for all classes, the videos were matched with individual data of accelerometers, so as to distinguish three types of sequences: warming up, inactive situations (e.g. teachers’ instructions or regulations of the lesson), and active periods. Warming up periods were not available for all the lessons videotaped, and were most of the time conducted collectively. Therefore, we compared entire lesson time (regrouping the three types of sequences together) *versus* active periods only in our analyses.

## 3. Results

Table 1 presents the descriptive statistics for PA-related behaviors (i) during the entire lesson and (ii) only during the activity periods, respectively. SED during activity periods are 15% lower than during the entire lesson, whereas light, moderate, and vigorous PA are proportionally higher during activity periods. We also note that standard deviation scores are higher when only activity periods are considered, compared to the entire lesson, indicating greater variability in individual students’ activity.

**Table 1:** Descriptive statistics (percentage of the time) for PA-related behaviors during the entire lesson and the activity-periods.

	Girls				Boys			
	Mini	Maxi	Mean	SD	Mini	Maxi	Mean	SD
Entire Lesson								
Sedentary	30.24	75.83	56.36	9.78	21.52	99.29	52.99	10.51
Light PA	10.93	35.27	20.56	4.94	0.24	34.63	21.10	4.68
Moderate PA	3.04	15.37	7.36	2.60	0.03	14.47	8.43	2.54
Vigorous PA	3.80	29.75	15.72	5.45	0.44	37.5	17.48	6.05
Activity period								
Sedentary	9.67	66.65	43.02	11.38	8.37	99.46	35.31	12.86
Light PA	10.93	35.27	20.56	4.94	0.22	55.13	29.09	7.32
Moderate PA	3.04	15.37	7.36	2.60	0.09	20.51	11.86	3.6
Vigorous PA	3.80	29.75	15.72	5.45	0.22	46.01	23.73	9.06

Table 2 presents the correlations between the scores of the different motivation constructs evaluated and students’ PA-related behaviors during the lesson. In the first four columns, those correlations were computed using the total duration of the PE lesson. In the last four columns of the table, only data from the activity periods are considered, that is, when students were supposed to be physically active.

**Table 2:** Correlations between motivational variables and PA-related behaviors (coefficients in bold are significant at the .05 threshold).

	Entire Lesson				Activity periods			
	Sedentary	Light PA	Moderate PA	Vigorous PA	Sedentary	Light PA	Moderate PA	Vigorous PA
Psychological needs								
Competence	**-.13**	.03	.08	**.19**	**-.19**	-.07	**.18**	**.24**
Autonomy	.00	.02	-.01	-.01	**-.12**	-.03	**.16**	**.13**
Relatedness	-.01	.00	-.03	.03	**-.19**	.01	**.15**	**.19**
Regulations								
Intrinsic motivation—stimulation	-.02	-.09	.00	.12	**-.16**	-.09	**.15**	**.23**
Intrinsic motivation—accomplishment	.01	-.09	.03	.07	**-.13**	-.05	**.12**	**.17**
Identified regulation	-.04	-.03	.07	.06	-.09	-.07	**.13**	**.13**
Introjected regulation—approach	-.10	.07	.07	.09	**-.11**	-.03	**.11**	**.13**
Introjected regulation—avoidance	-.10	**.15**	.05	.03	-.07	.02	.03	.07
External regulation—approach	**-.14**	**.14**	**.12**	.09	**-.13**	-.01	**.13**	**.14**
External regulation—avoidance	-.05	.11	.02	.00	-.02	.02	.01	.03
Amotivation	-.08	.12	.09	.01	-.02	.07	.00	-.02

### 3.1. Basic psychological needs and PA

We first looked at the correlations between the basic psychological needs and PA-related behaviors during the entire lesson. Results indicate that when the total PE time is taken into account, basic needs are unrelated to behaviors, with the exception of perceived competence that was negatively correlated with SED and positively correlated with vigorous PA. These relationships are in line with predictions and previous results; however evidence is scarce considering the number of correlations considered.

The pattern of results was quite different when considering the correlations between psychological needs and PA-related behaviors utilizing PA-related behaviors during only activity periods. Whereas only the competence need was related to PA-related behavior when considering the entire lesson sequence, it appears during activity periods that all three needs were related to PA. With the exception of light PA, we found significant correlations between all three needs and PA-related behaviors. Interestingly, SED was negatively associated with the three needs. Moderate and vigorous PA were positively associated with the three needs. Moreover, the needs of competence and relatedness were more strongly associated to vigorous PA than to moderate PA.

### 3.2. Self-determined motivation and PA

We looked at the correlations between motivations and PA-related behaviors in the entire lesson. Results indicate that when the total PE time is taken into account, overall self-determined motivations were unrelated to behaviors, with a few exceptions. Among the 32 correlations considered, only 4 were statistically significant. Only two forms of motivations among eight were related to PA behaviors; introjected-avoidance regulation was positively related to light PA, and external-approach regulation was positively related with light and moderate PA and negatively with SED. Vigorous PA was unrelated to any form of motivation.

The pattern of results was quite different looking at the correlations between motivations and PA-related behaviors when only activity sequences were considered. Among the 32 correlations tested, 14 were statistically significant. Five forms of motivations among eight were related to PA behaviors. Intrinsic motivation-stimulation and accomplishment were positively related to moderate and vigorous PA and for introjected-approach and external regulation-approach. Identified regulation was positively related to moderate and vigorous PA. Light PA was unrelated to any motivations.

## 4. Discussion

In this study, we investigated the relationship between motivation and PA-related behaviors in PE at the lesson level. Are PA-related behaviors related to motivation? Our results undoubtedly show that motivation is a significant correlate of PA-related behaviors in naturally occurring situations. Basic psychological needs were positively related to moderate and vigorous PA, whereas they were negatively related to SED. This result was confirmed by relationships between motivation and PA-related behaviors indicating that self-determined motivations (intrinsic and identified) and some non self-determined ones (introjected approach and external approach) were also positively related to moderate and vigorous PA and negatively to SED. These results are consistent with previous literature about the role of self-determination theory in PA-related behaviors, in particular in adolescents [20, 21]. Basic psychological needs and regulation types were both related to PA and SED outcomes. In particular, our results highlight the role of autonomous motivation while distinguishing the role of approach and avoidance orientation of controlled motivation, since avoidance-introjected and avoidance-external regulations were not related to PA behaviors. Few studies investigated this distinction in SDT literature [22, 23], and our study clearly demonstrated the importance of considering this distinction in showing how behavioral outcomes are differently linked to these regulations. More evidence in different contexts and with different outcomes (notably affective ones) would be necessary to fully assess this distinction and how it operates. Indeed, even if positive relationships between introjected and external regulations with PA behavior were found in our study, past research has largely supported the negative effects of these regulations on affective components [5].

Our results also bring new insights regarding methodological considerations that could lead us to better address the study of relations between motivation and PA-related behaviors in PE lessons. Indeed, one of the strengths of our study is the comparison of two different accelerometer measurement periods. Distinguishing and comparing between the entire lesson versus active-periods only enabled us to determine how sensitive the relationships between PA-related behaviors and students’ motivation really are. Indeed, it appears that the students’ individual PA-related behaviors were not well captured using entire lesson recordings. This is not such a surprise considering the traditional delivery of PE lessons and the amount of time where students act together, whether these situations are active (warming-up) or inactive (listening to teachers’ instructions). Traditional PE lessons are classically taught according to the following scenario: collective warming-up with all the students, learning core sequence of the lesson by group or individually (depending on the PA taught), and finally final feedback of information shared in front of all the students at the end of the lesson. Individual variability of behaviors appears to be captured in a more convincing way when we consider periods during which the student could act freely. This approach enables to disentangle the individual’s unique part of motivation from the noise induced in part by the structure of the lesson and by the teacher.

Additionally, our results are also informative of the specificity of light PA behavior in the PE context. Indeed, our results show that neither psychological needs nor motivations were related to this behavior. Future research relative to studying the relationships between motivation and PA-related behaviors will have to take into consideration the fact that the links between these two constructs are largely context-dependent, and isolating the individual PA-related behavior remains an open question in order to study this topic thoroughly.

This study is not exempt of limitations. First, it is mainly observational and therefore, from this work it cannot be inferred any link of causality between motivation constructs and children’s behavior during PE lessons. Thus, we could not manipulate neither the content of the lessons nor PE teachers’ teaching style (i.e. autonomy supportive vs controlled) to test our hypothesis. However, further studies could test it experimentally considering only active periods in order to understand better this relationship.

In conclusion, this study shed light on the importance of the link between psychological needs and motivation with PA related behaviors during PE lessons. Methodological considerations relative to the sequences of PA sessions considered in the analyses permitted to provide a meaningful support for motivational hypotheses during the active-only periods that was not found in the entire lessons recording.

## Conclusions

Linking psychological phenomena, such as motivation, to PA behavior in PE lessons was shown to be challenging depending on how the information recorded was computed. On previous studies, few links were observed between motivational constructs and PA patterns [8, 24]. Lonsdale et al. (2009), for example, compared differences in motivation between structured PE lessons and free choice periods. Stronger links were found between motivation and PA levels during free choice period compared to structured lessons, which could reflect the fact that more non-activity periods occurred during the structured lessons due to the interventions of the PE teacher. Our work shows that filtering those periods of non-activity during PE lessons is critical in order to properly compute the PA actually demonstrated by students during class time. Therefore, links found by Chanal et al. (2019) could have been weakened, not reflecting the real impact of autonomous motivation on trajectories of PA during childhood. Consequently, future works that aim to study the link between psychological constructs, such as motivation, and objective PA behavior should take this issue into consideration to avoid weakened results or misleading interpretations of the outcomes of their research.

## References

[1] World Health Organization. Global Strategy on Diet, Physical Activity and Health. Physical Activity and Young People. 2014.

[2] Dohrn I-M, Kwak L, Oja P, Sjostrom M, Hagstromer M. Replacing sedentary time with physical activity: a 15-year follow-up of mortality in a national cohort. Clin Epidemiol. 2018;10:179–86. 10.2147/CLEP.S151613 29416378 PMC5790069

[3] Hollis JL, Sutherland R, Williams AJ, Campbell E, Nathan N, Wolfenden L, et al A systematic review and meta-analysis of moderate-to-vigorous physical activity levels in secondary school physical education lessons. Int J Behav Nutr Phys Act. 2017;14. 10.1186/s12966-017-0504-0 PMC5402678 28438171

[4] Guay F, Ratelle CF, Chanal J. Optimal learning in optimal contexts: The role of self-determination in education. Can Psychol. 2008;49(3):233–40.

[5] Van den Berghe L, Vansteenkiste M, Cardon G, Kirk D, Haerens L. Research on self-determination in physical education: key findings and proposals for future research. Phys Educ Sport Ped. 2014;19:97–121.

[6] Ryan RM, Deci EL. Self-Determination Theory and the facilitation of intrinsic motivation, social development, and well-being. Am Psychol. 2000;55:68–78. 10.1037//0003-066x.55.1.68 11392867

[7] Shephard RJ. Limits to the measurement of habitual physical activity by questionnaires: Commentary. Brit J Sports Med. 2003;37:197–206. 12782543 10.1136/bjsm.37.3.197 PMC1724653

[8] Lonsdale C, Sabiston CM, Raedeke TD, Ha ASC, Sum RKW. Self-determined motivation and students’ physical activity during structured physical education lessons and free choice periods. Prev Med. 2009;48:69–73. 10.1016/j.ypmed.2008.09.013 18996143

[9] Johnson C. E., Erwin H. E., Kipp L., & Beighle A. Student Perceived Motivational Climate, Enjoyment, and Physical Activity in Middle School Physical Education. J Teach Phys Educ. 2017;36:398–408.

[10] Bryan CL, Solmon MA. Student Motivation in Physical Education and Engagement in Physical Activity. J Sports Behav. 2012;35:267–85.

[11] Owen KB, Astell-Burt T, Lonsdale C. The Relationship Between Self-Determined Motivation and Physical Activity in Adolescent Boys. J Adol Health. 2013;53:420–2. 10.1016/j.jadohealth.2013.05.007 23810429

[12] Erwin H. E., Babkes Stellino M., Beets M. W., Beighle A., & Johnson C. E. Physical Education Lesson Content and Teacher Style and Elementary Students’ Motivation and Physical Activity Levels. J Teach Phys Educ.2013; 32, 321–334.

[13] Gao Z, Zhang T, Stodden D. Children’s physical activity levels and psychological correlates in interactive dance versus aerobic dance J Sport Health Sci. 2013;2:146–151.

[14] Sebire SJ, Jago R, Fox KR, Edwards MJ, Thompson JL. Testing a self-determination theory model of children’s physical activity motivation: a cross-sectional study. Int J Behav Nutr Phys Act. 2013;10:111 10.1186/1479-5868-10-111 24067078 PMC3852537

[15] Cheval B, Courvoisier D, Chanal J. Developmental trajectories of physical activity during elementary school physical education. Preventive Medicine. 2016; 87:170–174. 10.1016/j.ypmed.2016.02.043 26946366

[16] Markland D, Tobin V. A Modification to the Behavioural Regulation in Exercise Questionnaire to Include an Assessment of Amotivation. J Sport Exerc Psychol. 2004;26:191–6.

[17] Li F. The exercise motivation scale: Its multifaceted structure and construct validity. J Appl Sport Psychol. 1999;11:97–115.

[18] Pelletier LG, Vallerand RJ, Sarrazin P. The revised six-factor Sport Motivation Scale (Mallett, Kawabata, Newcombe, Otero-Forero, & Jackson, 2007): Something old, something new, and something borrowed. Psychol Sport Exerc. 2007;8:615–21.

[19] Gillet N, Rosnet E, Vallerand RJ. Développement d’une échelle de satisfaction des besoins fondamentaux en contexte sportif. Can J Behav Sci. 2008;40:230–7.

[20] Owen K, Smith J, Lubans DR, Ng JYY, Lonsdale C. Self-determined motivation and physical activity in children and adolescents: A systematic review and meta-analysis. Prev Med. 2014;67:270–9. 10.1016/j.ypmed.2014.07.033 25073077

[21] Teixeira PJ, Carraça EV, Markland D, Silva MN, Ryan RM. Exercise, physical activity, and self-determination theory: A systematic review. Int J Behav Nutr Phys Act. 2012;30. 10.1186/1479-5868-9-78 PMC3441783 22726453

[22] Assor A, Vansteenkiste M, Kaplan A. Identified versus introjected approach and introjected avoidance motivations in school and in sports: The limited benefits of self-worth strivings. J Educ Psychol. 2009;101:482–97.

[23] Gagné M, Forest J, Vansteenkiste M, et al The Multidimensional Work Motivation Scale: Validation evidence in seven languages and nine countries. European Journal of Work and Organizational Psychology. 2015;24(2): 178–196.

[24] Chanal J, Cheval B, Courvoisier D, & Paumier D. Developmental relations between motivation types and physical activity inelementary school children. Psychology of Sport and Exercise. 2019; 43: 233–242.

